# The Effect of Ventilator-Associated Pneumonia on the Time-to-Extubation in Adult and Pediatric Intensive Care Unit Patients Requiring Mechanical Ventilation: A Retrospective Cohort Study

**DOI:** 10.7759/cureus.52070

**Published:** 2024-01-10

**Authors:** Raneem Turkistani, Afnan S Aghashami, Shatha S Badhduoh, Rowaida T Fadhel, Amani O Albaity, Israa A Malli, Sara Osman, Riyadh A Alshehri, Mona A Aldabbagh

**Affiliations:** 1 Medical Intern, King Saud Bin Abdulaziz University for Health Sciences College of Medicine, Jeddah, SAU; 2 Basic Medical Sciences, King Saud Bin Abdulaziz University for Health and Sciences College of Medicine, Jeddah, SAU; 3 Department of Pediatrics, King Abdulaziz Medical City, Jeddah, SAU; 4 Critical Care, King Abdulaziz Medical City, Jeddah, SAU; 5 Department of Pediatrics, Division of Infectious Diseases, King Abdulaziz Medical City, Ministry of National Guard Health Affairs, Jeddah, SAU; 6 Institutional Review Board, King Abdullah International Medical Research Center, Jeddah, SAU; 7 College of Medicine, King Saud Bin Abdulaziz University for Health Sciences, Jeddah, SAU

**Keywords:** prolonged mechanical ventilation, icu stay, time to extubation, intensive care unit, ventilator associated pneumonia

## Abstract

Introduction: Ventilator-associated pneumonia (VAP) ranks as a prominent hospital-acquired infection. VAP has been shown to have a detrimental impact on patients and healthcare facilities, leading to extended hospital stays, increased demands on medical resources, and higher financial expenses. This study aims to assess the influence of VAP on time-to-extubation and length of hospital stay (LOS) in patients requiring mechanical ventilation for more than 48 hours in pediatric and adult intensive care units (ICU).

Methods: This retrospective cohort study included adult and pediatric ICU patients admitted to King Abdul-Aziz Medical City in Jeddah, Saudi Arabia, from June 2016 to May 2020. The study encompassed ICU patients who required mechanical ventilation for more than 48 hours. Time-to-extubation and LOS were measured in days and compared between those who developed VAP and those who did not. A Kaplan-Meier curve was employed to estimate and compare both groups’ survival functions (time-to-event).

Results: The study involved 367 subjects, with 226 adults and 141 pediatric patients. Among the 367 mechanically ventilated patients, 33 (8.99%) developed VAP during their ICU stay, with 9 of them being children. VAP patients experienced a significantly longer time to extubation than non-VAP patients (13.5 vs. six days, p<0.0001). Likewise, ICU stays for VAP patients were significantly longer than those for non-VAP patients (19.5 vs. 13 days, p<0.002). However, the mortality rate at 28 days from intubation did not exhibit significant differences between the VAP and non-VAP groups (36.36% vs. 27.54%, p=0.283).

Conclusion: This study underscores that VAP patients face a substantial delay in time-to-extubation and an increased length of ICU stay compared to non-VAP patients. Such findings substantially impact the cost of hospital care and the risk of exposure to other infection-related complications while under mechanical ventilation. Enhanced preventive measures are warranted to reduce the occurrence and consequences of VAP

## Introduction

Patients in the intensive care unit (ICU) are at significant risk of developing hospital-acquired infections. Ventilator-associated pneumonia (VAP) appears at the top of the list of hospital-acquired infections that develop in patients intubated with mechanical ventilation for longer than 48 hours [1]. Hospital-acquired pneumonia/ventilator-associated pneumonia is the most common indication of carbapenem usage [2]. The incidence of VAP has remained stable for more than ten years, affecting approximately 10% of patients who are ventilated for more than 48 hours [3]. A study has documented the VAP rate in Makkah Region hospitals as 6.99 cases per 1000 ventilator days [4].

VAP is also associated with a significant increase in morbidity, mortality, antibiotics consumption, and health care costs. In addition, VAP can increase the time of hospital stay by two extra days and the risk of death by 14%, especially in those with comorbid conditions [5]. Another study on 6284 patients has shown that 13% of VAP-associated mortality resulted from prolonged ICU stays [6]. Many factors contribute to the increased risk of VAP, including patient characteristics, such as advanced age and male gender, increased mechanical ventilation time and prolonged length of hospital stay in supine horizontal positioning, comorbidities, prior antibiotic therapy, invasive operations, and contaminated respiratory equipment [7].

Tracheal extubation is a major step in patients’ recovery that helps decrease the risk of time-dependent complications associated with mechanical ventilation, including VAP. However, failure to extubate is associated with increased ventilation duration. Liberation failure is considered one type of extubation failure, and is defined as the inability to ventilate spontaneously without any mechanical aid, requiring reintubation within the first 24-27 hours [8]. According to Weinberg et al., upper airway obstruction and cardiac comorbidity have been identified as underlying causes of extubation failure and demonstrated a significant effect of VAP on extubation failure in their study [9].

Despite the significant impact of the preventive measures, VAP persists to be a cause for the death of critically ill patients in ICU, in addition to many factors that impede the quality of health provision. These difficulties can negatively affect the patient and the hospital, causing prolonged hospital stays due to delayed time-to-extubation and increasing demand for medical resources and financial expenses [7]. Improving the quality of the healthcare provided to VAP patients is a healthcare priority; thus, this study aims to evaluate the effect of VAP on time-to-extubation and length of hospital stay in adults and pediatrics ICU patients requiring mechanical ventilation for more than 48 hours in a span of five years from June 2016 till May 2020. Moreover, this study aims to to evaluate the effect of VAP on extubation failure and identify the causative pathogens associated with VAP.

## Materials and methods

This retrospective cohort study was conducted in King Abdulaziz Medical City in Jeddah, Saudi Arabia among ICU adults and pediatric patients to compare the time to extubation and length of ICU stay between VAP vs. non-VAAP patients. The study included all patients who required mechanical ventilation for more than 48 hours from June 2016 to May 2020. This study was done in intensive care units with 28 beds for adults and 14 beds for pediatrics. The study population included both adults (≥18 years) and pediatric (above 30 days - 17 years) ICU patients who required mechanical ventilation for more than 48 hours during the study period from June 2016 to May 2020. In this study, we excluded all pediatric patients with an age of 0-30 days, patients requiring mechanical intubation with a duration of less than 48h, patients who were initially chronically ventilated ''tracheostomized'', or patients with no available data.

The affirmative diagnosis of VAP was based on positive microbiological findings, new infiltrations in the radiological images, and increased oxygen requirements. The primary outcome of interest was time-to-extubation in days, calculated from intubation time. The secondary outcomes were the length of ICU stay in days for those who required mechanical ventilation beyond 48 hours, and 28 days mortality. The study was conducted at King Abdulaziz Medical City, and approved by the Institutional Review Board of King Abdullah International Medical Research Center, (JED-20-427780-56285 on 26 April 2020). Patient consent was waived due to the retrospective nature of this study and the research staff did not have any direct interaction with the participants in this research

Data Collection

A data collection sheet was utilized to collect information from the electronic (Best-Care) system, which consisted of demographic data such as gender and age, underlying medical condition, the development of VAP along with the ventilation settings, and the outcomes including time-to-extubation, LOS, and 28 days mortality. Data on extubation failure (reintubation within 48 hours since extubation) and the need for chronic ventilation (insertion of tracheostomy and need for ventilation for more than 28 days) were also congregated. Admission and discharge from ICU dates were obtained to calculate the length of stay at the ICU by counting the days in Microsoft Excel v16.0. Similarly, intubation and extubation dates were also collected to calculate the time-to-extubation by the same method. 

Data Analysis

Categorical data were described by frequencies and percentages and compared using the Chi-square test. Numerical data were analyzed using non-parametric measures such as median, minimum, and maximum. The Mann-Whitney test was used to determine the relationship between the numerical data. Length of time data such as time to extubation and length of ICU stay were analyzed using the time to event analysis and Kaplan-Meier curve. Statistical analysis was performed using JMP software (John’s Macintosh Project), version 10.0 (SAS Institute Inc., Cary, NC, USA).

## Results

This study included 367 patients who were mechanically ventilated for more than 48 hours and met the inclusion criteria. Of all subjects, 226 (61.58%) were adults, and 141 (38.41%) were pediatric patients. During the ICU stay, VAP was confirmed in 33 subjects (8.99%). A comparison between VAP patients’ and non-VAP patients’ demographics is summarized in Table 1. There was no difference in the median age between the VAP and non-VAP groups, 45 vs. 48 years (p=0.542), and there was no difference in the rate of VAP in adults compared to pediatric patients (10.6% vs. 6.8%; p=0.167). The most commonly reported underlying medical condition in both VAP and non-VAP cases were respiratory diseases, accounting for 21.21% and 24.58%, respectively. On the other hand, there were significantly more people who had trauma as an underlying condition in the VAP group compared to the non-VAP (18.18% vs. 5.69%; p= 0.006).

**Table 1:** Descriptive Statistics Comparing VAP to Non-VAP ^ǂ ^using Mann-Whitney with Cl 95% ^ǀ^ using the chi-square test with Cl 95% ^*^Using the Fisher exact test with a CI of 95% NA: Not applicable.

Variables	VAP patients	Non-VAP patients	p-value
	n=33	n=334	
Median age in years (min-max)	45 (0.08-89)	48 (0.08-96)	0.542^ǂ^
Age groups (%)			
Adults	24 (72.73)	202 (60.84)	0.167^ǀ^
Pediatrics	9 (27.27)	132 (39.52)	
Gender (%)			
Male	21(63.64)	211(63.17)	0.958^ǀ^
Female	12(36.36)	123(36.83)	
Underlying disease specialty (%)			
Cardiology	3 (9.09)	17 (5.09)	0.407^*^
CNS Disorders	2 (6.06)	55 (16.47)	0.135^*^
Dermatology	1 (3.03)	1(0.30)	0.172^*^
Endocrinology	1 (3.03)	3 (0.90)	0.315^*^
ENT Disorder	0	3 (0.90)	NA
Gastroenterology	1 (3.03)	18 (5.39)	1^*^
General Pediatrics	2 (6.06)	4 (1.20)	0.093^*^
Hematology	1 (3.03)	6 (1.80)	0.485^*^
Hepatology	0	2 (0.60)	NA
Immunology	0	1 (0.30)	NA
Infectious Diseases	3 (9.09)	36 (10.78)	1^*^
Nephrology	0	12 (3.59)	NA
OBGYNE	0	1 (0.30)	NA
Oncology	6 (18.18)	61 (18.26)	0.99^ǀ^
Orthopedics	0	8 (2.40)	NA
Respirology	7 (21.21)	83 (24.58)	0.643^ǀ^
Trauma	6 (18.18)	19 (5.69)	0.006^ǀ^
Pediatric surgery	0	1(0.30)	NA
Vascular surgery	0	1(0.30)	NA

A comparison of clinical outcomes in the VAP and non-VAP groups is summarized in Table 2. VAP patients had a significantly longer time-to-extubation than non-VAP patients (13.5 vs. 6 days, p<0.0001). Similarly, the length of ICU stay in VAP patients was significantly higher than in non-VAP patients (19.5 vs. 13 days, p<0.002). The extubation failure in patients in the VAP group was higher than in the non-VAP group, but this did not reach statistical significance (24.24% vs. 14.48%, p=0.062). Likewise, there was no difference in the 28-day mortality rate from the intubation time between the VAP and non-VAP groups (36.36% vs. 27.54%, p=0.283).

 

**Table 2:** Descriptive Statistics Comparing Clinical Outcomes of VAP Patients and Non-VAP Patients ^ǂ^ using Mann-Whitney ^ǀ^ using the chi-square test

Variables	VAP patients n=33	Non-VAP patients n=334	p-value
Median Time to Extubation (min-max)	13.5 (7-22)	6 (2-28)	<0.0001^ǂ^
Median Length of ICU Stay (min-max)	19.5 (9-178)	13 (2-316)	0.002^ǂ^
Extubation failure (%)	8 (24.24)	42 (14.48)	0.062^ǀ^
Outcomes (%)			
· Death in 28 days	12 (36.36)	92 (27.54)	0.283^ǀ^
· Chronic Ventilation	12 (36.36)	66 (19.76)	0.026^ǀ^

**Figure 1:**
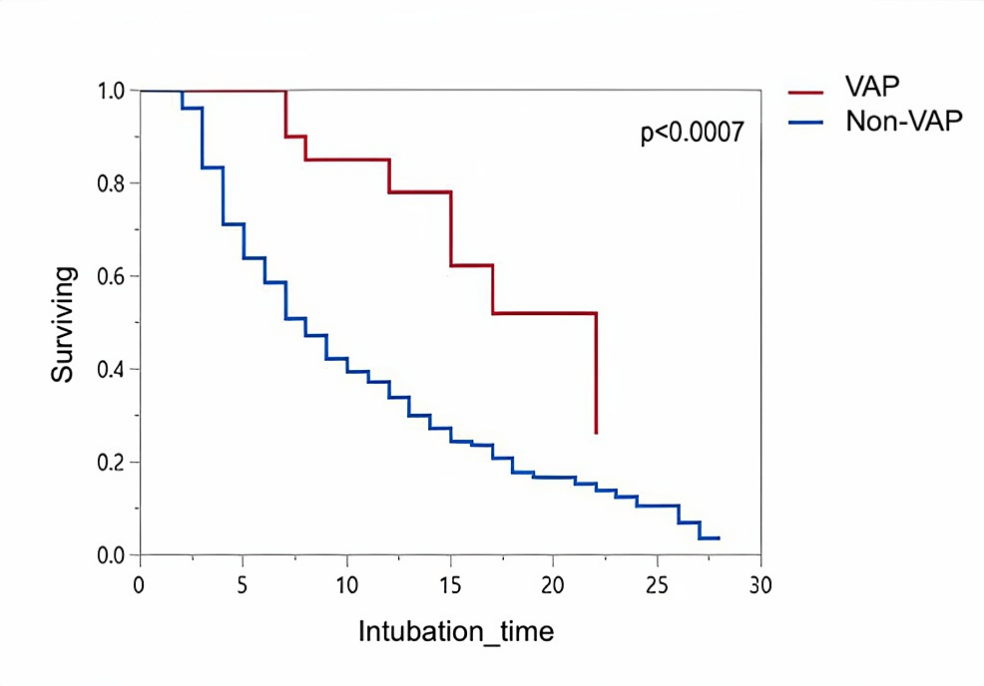
Kaplan-Meier curve demonstrating time to extubation in VAP and non-VAP patients

**Figure 2:**
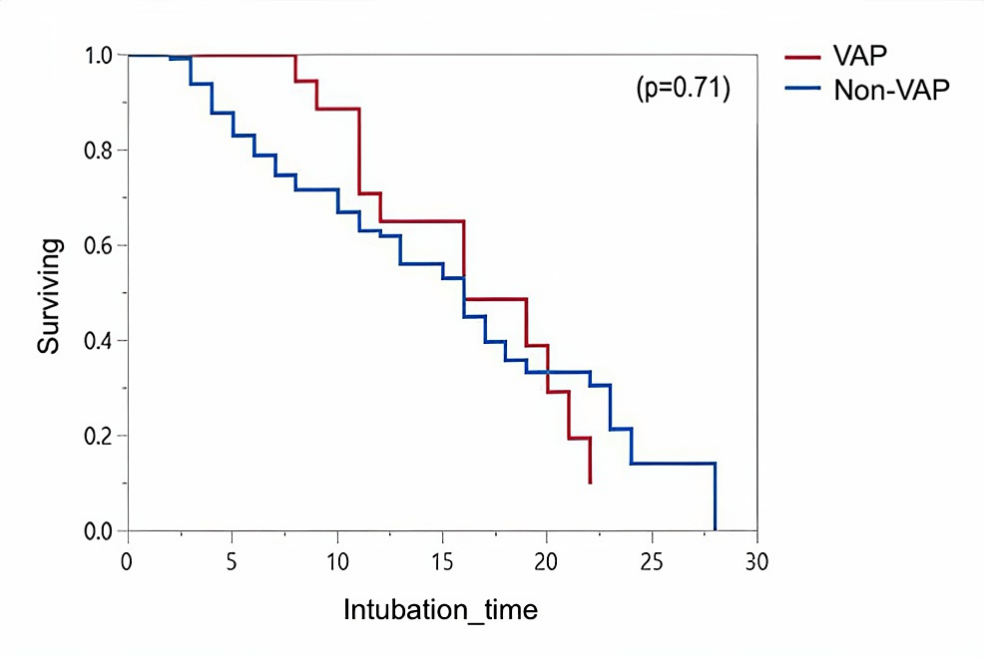
Kaplan-Meier curve illustrating mortality in VAP and non-VAP patients within 28 days since intubation.

A Kaplan-Meier curve demonstrates time-to-extubation in patients with VAP and non-VAP in Figure 1. Extubation time is significantly delayed in VAP patients compared to non-VAP (p<0.0007). Mortality of VAP and non-VAP groups within 28 days since intubation is also illustrated in Figure 2. The intubation time maximum is set to 28 days and patients with a longer intubation time were excluded. The median time from intubation to death in VAP was not different from the non-VAP (p=0.71). All pathogenic isolates identified as the cause of VAP in this study are summarized in Table 3. Gram-negative organisms constitute 87% of all isolates, with Klebsiella Pneumonia as the most common agent at 23.07%, followed by Acinetobacter baumannii in 15.38% of the cases. In addition, 25 (64%) of the identified pathogens were multi-drug-resistant

**Table 3:** Description of pathogenic isolates identified in VAP cases ^∞^: Numbers are not additive; some patients had more than one pathogen identified *: Multidrug-resistant (MDR) isolates ^£^: Six isolates are MDR ^¥^: Three isolates were Methicillin-resistant Staphylococcus aureus ^€^: Two isolates were MDR ^(%)^: calculated by dividing the number of isolates per category by the total number of identified organisms

Respiratory isolates^∞^	Number (%)
Klebsiella pneumonia^£^	9 (23.07%)
Acinetobacter baumannii*	6 (15.38%)
Stenotrophomonas maltophilia*	5 (12.82%)
Staphylococcus aureus^¥^	5 (12.82%)
Pseudomonas aeruginosa^€^	4 (10.25%)
Serratia marcescens*	3 (7.69%)
Haemophilus influenza	2 (5.12%)
Elizabethkingia meningoseptica*	2 (5.12%)
Enterobacter cloaca*	1 (2.5%)
Burkholderia pseudomallei	1 (2.5%)
Escherichia coli	1 (2.5%)

## Discussion

VAP persists to be a common infectious complication among critically ill patients [10]. This study revealed an incidence of VAP of 8.99%. In addition, the study found that VAP-affected patients had a significantly longer time to extubation than non-VAP patients. Similarly, VAP played a major role in the length of ICU stay and extended the duration of hospitalization. However, this did not imply mortality.

These findings match the results of a study done by Papazian and colleagues where patients who acquired VAP had a median ICU stay of 21 days and received mechanical ventilation for a median duration of 15 days, while patients who did not acquire VAP had a median ICU stay only seven days [11]. Similarly, in another study by Al-Dorzi et al., time-to-extubation in VAP patients was significantly higher compared to non-VAP (19.3±18.0 vs 8.9 ± 8.8 days, respectively, (p<0.001), which led to a prolonged ICU (22.2 ± 19.2 vs 10.7 ± 9.0 days, respectively, p<0.001) and hospital stay (85.5 ± 93.3 vs 61.6 ± 87.0 days, respectively, P<0.001) [10]. The extubation failure in patients in the VAP group was also higher than in the non-VAP group but not statistically significant. A recent study reported that the 30-day mortality rate after the diagnosis of VAP among adults was 30%, which is compatible with our study [12]. A meta-analysis of 6284 individual patient data from 24 VAP prevention trials has revealed a 30% overall mortality rate of 848 adult VAP patients, comparable to our study [6]. The mortality rate was attributed to prolonged ICU stays and its possible complications, such as those related to invasive procedures or additional nosocomial infections. VAP did not contribute to additional ICU days and its attendant mortality in severely ill patients with expected prolonged ICU stay [6].

A systematic review by Arabi and colleagues on the incidence of VAP in developing countries demonstrated that the VAP rate was higher in developing countries compared to the National Healthcare Safety Network (NHSN) benchmark rates [12-14]. This higher rate may be attributed to various reasons, including lack of expertise, insufficient medical resources and modern technologies, inadequate nurse-to-patient staffing ratio, overloaded ICUs, and poor compliance with the guidelines [15-16]. Respiratory illnesses were the most common underlying disease associated with ventilation, while trauma patients have significantly higher chances of developing VAP. According to Arumugam et al. 2018, higher injury severity indicators such as a lower Glasgow Coma Scale and higher abbreviated injury score, presence of head and chest injuries, and advanced age in trauma patients are important risk factors of VAP development [17].

The study intended to recognize the causative pathogens associated with VAP and identified that gram-negative organisms, specifically *Klebsiella pneumoniae*, were the most common pathogen causing VAP, followed by *Acinetobacter baumannii*. At the same time, *Staphylococcus aureus *was a commonly reported gram-positive organism in patients with VAP. Earlier studies reported that *Pseudomonas aeruginosa* (up to 52%) was the most common causative agent for VAP, followed by *Acinetobacter spp* (up to 36%) [13]. Recent local data confirmed the rise of multidrug resistance in subjects with VAP, with *Acinetobacter baumanii *as the most prevalent multi-resistant pathogen, followed by *Pseudomonas*, MRSA, and *Klebsiella spp* [18]. These organisms are major contributors to several hospital-acquired infections and are well known for their ability to accumulate and transfer drug-resistant determinants [19]. The majority of VAP causative agents in our ICU were caused by multidrug-resistant organisms, which warrants undertaking extensive infection control measures.

Various strategies can be grouped and applied to form a bundle of multidisciplinary medical practices to decrease VAP incidence. The implementation of a VAP prevention bundle, such as head elevation, oral hygiene, and sedation interruption, was examined in many studies [20]. In the study by Bigham et al., VAP bundle application reduced the VAP incidence rate from 5.6 to 0.3 per 1000 days on a mechanical ventilator (p<0.0001) [20]. Similarly, another study by De Cristofano and colleagues found that bundle application decreased the VAP rate from 13.2% to 4.5% (p=0.0047) [21]. On the other hand, Osman et al. conducted a study to investigate the effect of VAP prevention bundle pre-and post-implementation at the pediatric intensive care unit (PICU) of King Abdulaziz Medical City (KAMC), Jeddah, Saudi Arabia, from January 2015 to March 2018. The study reported that 18 ventilator days per 1000 of the pre-bundle group had developed VAP, which was 4% more than the bundle group. The VAP bundle did not significantly reduce the VAP rate in the PICU [22].

Limitations and Recommendations

This is one of the few studies done in Saudi Arabia measuring the effect of VAP on the time-to-extubation and length of ICU stay with time-to-event analysis. Our research was conducted in a single center and was hindered by the missed/ limited extubation failure data and the limited sample size. The retrospective cohort study design also limited the ability to consider some confounding factors. This emphasizes the need for future multicenter studies that are conducted prospectively with a larger sample size and control for confounding factors. 

## Conclusions

In conclusion, this study has focused on the effect of ventilation-associated pneumonia on the time of extubation and length of intensive care unit stay for patients requiring mechanical ventilation for more than 48 hours. Other ventilation-associated infections, such as ventilator-associated tracheitis, were not investigated. Our time-to-event analysis has shown that adult’ and pediatric’ ICU VAP patients have a significant delay in time-to-extubation and increased length of ICU stay. In addition, Gram-negative organisms were the most common causative pathogens, specifically *Klebsiella pneumoniae*, associated with VAP in our study. Such findings would have a major impact on increasing the cost of hospital care and the risk of exposure to other complications related to infections and being under mechanical ventilation. Thus, immediate preventive measures are needed to prevent VAP and its consequences.
